# The Relative Value of Symptoms

**Published:** 1895-06

**Authors:** Samuel A. Kimball

**Affiliations:** Boston, Mass.


### THE RELATIVE VALUE OF SYMPTOMS.

SAMUEL A. KIMBALL, M. D., BOSTON, MASS.

[Read before the Bœnninghausen Club, May 9th, 1895.]

MEMBERS OF THE BŒNNINGHAUSEN CLUB:—When we have before us on the one hand the symptoms of a carefully-examined patient and on the other the provings of our materia medica, what method of procedure shall be followed in order to find the remedy most suitable to the case? What value is to be placed upon the various symptoms? Which are to be considered of more significance than others and why should some be most essential in the selection of the remedy and others only of secondary importance? Every case presents such questions for consideration and their solution is often difficult.

Section 18 of the *The Organon* reads as follows:

"From this indubitable truth, that besides the totality of the symptoms nothing can by any means be discovered in diseases wherewith they could express their need of aid, it follows undeniably that the sum of all the symptoms in each individual case of disease must be the sole indication, the sole guide to direct us in the choice of a remedy."

Also section 153: "In this search for a homœopathic specific remedy, that is to say, in this comparison of the collective symptoms of the natural disease with the lists of symptoms of known medicines, in order to find among these an artificial morbific agent corresponding by similarity to the disease to be cured, the *more striking, singular, uncommon, and peculiar* (characteristic) signs and symptoms of the case of disease are chiefly and almost solely to be kept in view; for it is *more particularly these, that very similar ones in the list of symptoms of the selected medicine must correspond to*, in order to constitute it the most suitable for effecting the cure. The more general and undefined symptoms: loss of appetite, headache, debility, restless sleep, discomfort, and so forth, demand but little attention when of that vague and indefinite character, if they cannot be more accurately described, as symptoms of such a general nature are observed in almost every disease and from almost every drug."

There is, apparently, a contradiction between the statement in section 18 that "the sum of all the symptoms must be the sole indication, the sole guide in the choice of the remedy," and that in section 153, that "the more striking, singular, uncommon, and peculiar (characteristic) signs and symptoms are chiefly and almost solely to be kept in view," but upon closer study this is apparent only and not real.

In stating that the totality of the symptoms must be the sole indication to direct us in the choice of the remedy Hahnemann undoubtedly intended to emphasize the idea that we are not to give weight to hypotheses in diagnosis or to indulge in any theoretical speculations in regard to the case, but to confine ourselves solely to the subjective and objective facts presented to us by the patient.

In this way the totality of the symptoms is to be the only *source* of our indications for the selection of the remedy.

But as the symptoms vary in value from the general conditions present in almost every case of illness to the particular symptoms of the case to be considered, it was necessary to point out what symptoms are of most importance in this selection, and this is shown in the statement, that the most striking, singular, uncommon, and peculiar symptoms are chiefly and almost solely to be kept in view, or, in other words, the symptoms that are peculiar to and individualize this case from other cases of the same disease or other diseases, not the diagnostic symptoms, but those peculiar to the patient and not to the disease.

It must not be expected from this that in every case we will find some striking, singular, uncommon, or peculiar symptom to guide us to the curative remedy. Such symptoms are exceptional rather than the rule; we do not find them often, but we do find symptoms not particularly striking, uncommon, or peculiar and yet, not being necessary to the diagnosis, they are individual to, or characteristic of the case in question.

These symptoms of themselves may be of no special value, but become valuable or characteristic by their conditions of aggravation or amelioration, their concomitants or locality.

Neither is a given symptom always a characteristic; it may be markedly so in one patient and in another a general symptom only. An aggravation or amelioration may alone be the characteristic symptom of a case, as aggravation from drinking milk, or amelioration from being carried slowly.

Giving undue value to a characteristic has led to prescribing upon single symptoms only, an idea taken from the "key-note" system of Dr. Guernsey, which has been much abused and misunderstood. In his preface to the first edition of his *Obstetrics*, Guernsey states that by his key-note system he means to take some strong characteristic symptom, which will often be found to be the governing symptom of the case and on referring to the materia medica, the other symptoms will be found there also under the same remedy. Hering objected to this on the ground that all of our most approved characteristics are always found under more than one drug, and Lippe feared that regarding single characteristics of so much importance would lead to the habit of prescribing upon one prominent symptom alone.

The characteristics are important, and the tendency to depend upon them alone may insensibly be acquired; they should be considered only, however, as *indices* to the remedies that are to be studied in the materia medica, and used in this manner they are of the greatest value.

Symptoms upon which we base our diagnosis are of least importance in the selection of the remedy, since they are common to all such diseases. But in the absence of other symptoms, and this often happens, they may be all we have to guide us, and then, with their aggravations, ameliorations, and concomitants, they become the leading symptoms of the case.

Cases of dysentery are accompanied by tenesmus before, during, or after stool—that is one of the important diagnostic points, and, with the bloody mucous stool, fever, and other general symptoms may be all that is presented to us.

Now simple tenesmus is common to so many remedies that in itself, it is of no value in selecting the remedy, but if attended with conditions of aggravation or amelioration or with concomitants, it may become the leading indication, for instance, in Nux-vomica the tenesmus and the pains in the back cease with the stool; in Mercurius and Capsicum they continue after it.

In section 153 quoted above, Hahnemann says: "The more general and undefined symptoms: loss of appetite, headache, debility, restless sleep, discomfort, and so forth demand but little attention when of that vague and indefinite character, if they cannot be more accurately described."

But if they can be more accurately described by conditions or concomitants, they may be the only symptoms in the case by which to prescribe.

Generally speaking, functional symptoms of an affected organ are of much less value than symptoms which occur in other parts during the exercise of the function of that organ.

Burning pain in the urethra during or after micturition is of little value in gonorrhœa, for it is usually present, but pain in the testicles, thighs, or abdomen during or after micturition, or symptoms of some other part not immediately concerned in that function would be more important.

So also pain in the stomach after eating, in an attack of indigestion, is not of as much value as vertigo or headache after eating would be in the same attack.

Therefore symptoms that affect the general organism are of more value than those that are functionally related to the organ affected.

Symptoms also that affect the patient's general condition, such as the effects of heat, cold, drafts, etc., are of more importance than transient symptoms, even if they should be odd or peculiar.

The symptoms in a cured case disappear in the inverse order of their appearance, the symptoms recently developed disappearing first, the oldest ones last. The more recent symptoms are valuable as being the latest expression of the diseased condition and must be covered by the remedy. This is so when a second or a new remedy is to be given, the last symptoms that appear must be the guide to it. So also after the favorable action of a remedy which seems to have accomplished all it can, and there are still symptoms remaining that call for treatment, then, as the symptoms come and go, if a new symptom appears it will be of great value, and will probably decide the choice of the next remedy.

If, after a remedy has been given, new symptoms appear, which are not in the history of the patient, they will very often be found in the pathogenesis of the last given drug, and, if the patient generally improves, the new symptoms usually pass away and are unimportant; but if they persist and there is no general improvement, it was a wrong selection, and they are of first importance in the choice of a new remedy.

In giving such a high position to the latest developed symptoms, the older symptoms must not be neglected, especially those that were the first indications of a departure from health. These are of the greatest value, particularly those occurring before there was any treatment or any metastasis from palliative or maltreatment.

Such symptoms are the first expressions, possibly, of an internal chronic miasm, and must be covered by the remedy as nearly as can be done. The difficulty in obtaining such expressions may often account for our failure to make a satisfactory selection.

The reappearance of old symptoms indicates a curative action of the remedy if they appear in the inverse order of their development, and are valuable indications that the vital force is overcoming the diseased condition. They must be carefully let alone unless they are so severe as to retard the general improvement.

If there is an aggravation of existing symptoms after the administration of a remedy, which is of short duration, and there is an improvement generally, it is a curative effect, and the aggravation must be respected, and not interrupted; but if the aggravation continues and the general state of the patient is worse, then the aggravated symptoms are important, and the condition must be corrected by antidoting the last-given remedy. Possibly this may be done by a different potency of the same remedy, or in case no antidote is known it may be necessary to make a new prescription, giving the highest value to the aggravated symptoms.

Symptoms are valuable that show a disease is getting better from within outward, from above downward, from the more essential to the less essential parts. Such symptoms indicate an improved condition, and must not be interfered with; as the disappearance of internal symptoms and the appearance of an eruption upon the skin, together with a general improvement in the patient.

When, on the contrary, symptoms show that the diseased condition is advancing from without inward, from below upward, from the less essential to the more essential parts, these are danger-signals that must be respected; as the disappearance of eruptions followed by serious brain symptoms, or the metastasis of rheumatic symptoms from the extremities to the region of the heart, then these new symptoms are of the utmost importance, and show the necessity for a new prescription.

In regard to aggravations and ameliorations, those which stand out in marked contrast to the general condition of the patient are the ones to be chiefly considered. If a pain is aggravated only when lying on the back, and the patient is comfortable in every other position, either lying on the side, sitting, standing, or walking, the aggravation from lying on the back is the important symptom and the one to receive most attention.

Or, if a pain is ameliorated only when lying on the back, and there is much discomfort in every other position, these aggravations are not to be considered, but the condition in which the patient is better, the amelioration from lying on the back as distinguished from the generally aggravated condition of the patient is the symptom that is most valuable.

It is necessary for us to be thoroughly acquainted with our pathology and diagnosis in order that we may not be misled into giving more importance to certain symptoms than is justifiable, or into overlooking others from a lack of knowledge to distinguish what pertains to such diseases in general and what is individual to the particular case under consideration.

When we come to the consideration of acute and chronic cases, the symptoms resolve themselves into two classes, subjective and objective, both important, both often seen in the same case, yet either may be the sole method of expressing the diseased condition.

Of the two the objective symptoms, when not diagnostic of a pathological condition are probably the more important. The subjective symptom is the description by the patient of his feelings as they appear to him, but sick people differ much in their ability to express themselves, and it is an easy matter to be misled by the wrong interpretation of a sensation. It is perplexing for some people to tell how they feel at the present time, and almost impossible for them to describe the sensations of two or three days ago, but with others it is difficult to stop the flow of symptoms when once started, and equally so to differentiate the essential from the non-essential.

With the objective symptom it is different. Here is a positive, almost involuntary expression on the part of the patient. If we are called to a case and the patient suddenly doubles up with pain and pressing hard against the abdomen with his hands or any other object, moans and groans for a few moments, then sinks back with relief, and in a short time repeats the performance, we would not care much whether his intestines felt as if they were being squeezed between stones or telegraph poles, the object lesson of his movements and positions would probably be all-sufficient.

With those who have no other method of expressing their uncomfortable sensations the objective symptoms are, of course, most important. In young children they are all we have, and much can be learned from them, such as, the general appearance of the child, the expression of the face, wrinkles on the forehead, dilatation of the alæ nasi, regurgitation of food, or vomiting, time and character of the stools, appearance of the urine, and so on.

Important as symptoms are when the child is awake, those occurring during sleep are even more so. The system being entirely relaxed, the sick child then shows most important indications for the remedy : the position of the body, movement of the limbs, twitching of various muscles, moaning, whining or crying out. Then on waking, is the child good-natured or irritable, or does it wake with fear and terror?

Objective symptoms also are our only reliance in the treatment of animals. In acute cases the subjective symptoms are ordinarily so pronounced that there is not much danger of deception, and the objective symptoms, when present, are equally prominent. In mental conditions the more serious they are the more important are the objective symptoms. This is especially so in cases of mania or unconsciousness.

In one case of mania there was absolute refusal to talk or to answer questions; food was refused; there was sleeplessness, retention of urine, and a profuse warm sweat all over; there was a suspiciousness or fear, with pulling of the bed-clothes up to his face and peering over them; there were some attempts to strike, and a general feeling of hostility toward everything and everybody. A solution of Belladonna$^{200}$ was made at 10 P. M., and a handkerchief, moistened with it, was held over his nose for several inhalations. In twenty minutes after the inhalation he slept for half an hour, and on waking took a little milk. Soon he slept again—for two hours, and took more nourishment; then slept the remainder of the night, and in the morning was quiet and rational.

We see the importance of objective symptoms, also, in cases like the following: A lady seventy years of age had an attack of acute bronchitis, with considerable rattling of mucus and pain in the chest on coughing. Bryonia did not relieve, and then a drowsiness came on, with a loose, rattling cough, little expectoration, more or less nausea, and a temperature of 103. Antimonium-tart. caused no change, and the next day she was decidedly worse. There was loud breathing, with puffing of the cheeks on expiration, mouth open, with snoring at times, involuntary urine, a red, dry tongue, moaning and muttering in sleep, the head much confused, and with difficulty could she be roused to answer questions. Three doses of Opium$^{45m}$ (Fincke) were given in water an hour apart, and the next day she was rational, the temperature normal, and she quickly recovered.

Much information is obtained by the careful observation of a patient, and this can be cultivated by practice to an extent not thought of by a beginner. The most valuable symptoms are seen when the patient is sleeping or when unconscious of observation, then the system is relaxed and the symptoms shown are the natural expressions of the patient's feelings.

The subjective symptoms often depend largely upon their aggravations and ameliorations for their value, or the aggravation or amelioration itself may be the important fact. The objective symptoms are not usually so dependent upon their conditions.

In a diphtheria the side upon which it begins may decide the remedy; sometimes the appearance of the membrane, but rarely without confirmatory symptoms. The aggravation or amelioration from swallowing hot or cold drinks, however, may be decisive.

In diseases of the chest, coughs, pneumonias, etc., we find the subjective and objective symptoms of about equal importance, but in these conditions, as in all others, the value of the symptom depends less upon its diagnostic importance than anything else. Is the cough aggravated in the open air or in the warm room? Does it seem to come from the stomach, and are there sharp pains through the chest to the back? Is the patient aggravated or ameliorated when lying on the affected side in a pneumonia, or is the plueritic pain better sitting up or lying down? Any one of these symptoms may decide the choice of the curative remedy.

In diseases of the digestive organs, such as indigestions, diarrhœas, dysenteries, we have the same combination of subjective and objective symptoms, varying in importance in different cases, and we must always endeavor to obtain a description of the most trivial sensation, and pay attention to the slightest action of the patient.

The diagnosis is not to be thought of in the selection of the remedy, except that when two symptoms are compared the one having less to do with the diagnosis is usually the more important, although the aggravation or amelioration of a diagnostic symptom may constitute it the most valuable one in the case.

In attaching small importance to diagnostic symptoms in the choice of the remedy we must not forget to give them their rightful position in the general management of the patient.

It is extremely important that a correct diagnosis be made, or at least that we know the general tendency of the disease, the organs affected, and what will be the probable outcome.

We must know by examination of the chest whether or not it is safe for a patient recovering from pneumonia to go out; by examination of the urine the condition of our patient with diabetes or diseases of the kidneys, and be qualified to make necessary investigations in other diseased conditions. The character of the disease also determines to a large extent the question of diet, which is most important.

In the treatment of chronic diseases it seems as if less distinction were made between the diagnostic symptoms and those peculiar to the individual, possibly because they do not stand out so sharply as in acute diseases, and we are apt to take the symptoms down without giving as much attention to the diagnosis.

The same rules hold, however, here as in acute diseases in regard to the value of diagnostic and non-diagnostic conditions.

When we have a carefully examined chronic case with all its numerous symptoms, what value is to be given them, how and where are we to begin in our search for the remedy? The latest developments are of course important, and the earlier ones no less so. We often find in tracing back the psoric manifestations, that they began as sick headaches, indigestion, diarrhœa, or some other acute attack, and the careful description of these early symptoms, what made them better or worse, their concomitants and locality, are most important.

All the conditions of childhood must be considered: a moist or dry eruption on the head when a baby, was he fat or thin, were there swollen glands, was he poisoned with vaccine virus?

A lateness in the first menstruation is important, or any disturbance at that time, all conditions of menstruation, if abnomal, are valuable. In this manner we must investigate the examination of the patient, which should extend from his earliest recollection to the present time, and consider carefully what is presented. Nor must we slight the examination of his present condition. We may find valuable indications in the mental sphere; a suicidal tendency, fears of various things, worse when alone or in company, aggravations in the dark, desire for or aversion to light.

There may be craving for certain articles of food, or aversion to them, or an aggravation from some particular thing, as milk or acids.

Faintness at the stomach at certain hours of the day or night. Conditions of aggravation or amelioration of hæmorrhoids, cracking of the joints, sweat of the hands or feet, the various positions in sleep, the effect of heat or cold, aggravation from the heat of the sun, or from drafts, aggravation from jarring, or from riding in a wagon, craving for open air. There may be a history of warts, cracks, chilblains, corns, gouty nodes, moles.

Any one of these or similar symptoms may be the starting point in the search for the remedy; possibly a peculiar or uncommon symptom, or one made important by its aggravations, ameliorations, concomitants, or locality.

An aggravation or amelioration alone may be the most valuable symptom in the case. Diagnostic symptoms must not be overlooked, especially when attended with concomitants or conditions; neither must functional symptoms of the organ affected be neglected, although these, with diagnostic symptoms, usually take a secondary place in the selection of the remedy, when other symptoms are present.

In the treatment of acute exacerbations of chronic diseases and in the acute miasmatic diseases, also, we often find that the patient in his present condition does not give valuable indications for a remedy; the symptoms are general and not particularly indicative, or else with fairly good indications there is no response to careful prescribing.

Then it is necessary to go back and prescribe for the constitutional symptoms that were present before the attack came on, and possibly to return to the conditions of years before for a suitable remedy.

After the remedy has been given what value shall be placed upon the symptoms that then appear, how shall they be considered in relation to the last remedy prescribed or to those yet to be selected?

It has been well said that the difficulties of a case begin after making the first prescription. This is especially so in chronic cases, and it may not be amiss to briefly state again the conditions that may arise.

If there is amelioration of the symptoms or a return of old symptoms nothing is to be done. Old symptoms as they come and go are not to be interfered with unless they interrupt the improvement, and the general condition of the patient is worse and the cure retarded. Even then great caution must be used in repeating the same remedy or making a new selection.

Aggravation of existing symptoms and general improvement mean no interference, but with an aggravation that is continued, the general condition of the patient being worse, then the remedy must be antidoted or a new prescription made, the aggravated symptoms being the most important indications. If new symptoms appear, that have not been experienced before by the patient, with a general improvement, they usually pass away, but if they persist and there is lack of improvement they are of great value in a new selection.

Symptoms that show a general improvement from within outward, from above downward, from the more essential to the less essential parts, are to be respected and not interfered with by a new prescription; but symptoms that go in an opposite direction, showing that the diseased condition is progressing from without inward, from below upward, from the less essential to the more essential parts, these must be taken as guides to the selection of a new remedy.

In general, it may be said that the most valuable symptoms that appear after a prescription are the aggravations that persist without general improvement, the new symptoms that appear with the same conditions of persistence and lack of improvement, and the symptoms that show a change for the worse in their metastases from place to place. In a curable case, these last must be promptly met, but in an incurable disease the extent of the suffering may determine the necessity for a new prescription, as in such cases the most that can be hoped for is as comfortable a release as possible.

When two or three of the chronic miasms are present—psora, syphilis, and sycosis—Hahnemann tells us to first prescribe for the psoric miasm, then for the sycotic, and lastly for the syphilitic.

This presupposes the ability on our part to differentiate between psoric, syphilitic, and sycotic symptoms, which would be a most valuable acquisition if true; but the subject demands a more extended treatment than is possible to give it here.

It may be said, however, that all men, and women also, are psoric. There may be a few exceptions where several generations have been under strictly homœopathic treatment; but in a general way, we are all more or less psoric, possibly more or less sycotic and syphilitic also. Who knows?

At all events, the sycotic and syphilitic symptoms are probably engrafted upon a psoric base, so that we would be safe in beginning with an antipsoric, unless there were stronger indications for another class of remedies. Many remedies may at the same time combine symptoms of two of the miasms, and a few may combine all three; different groups of remedies may be more psoric, sycotic, or syphilitic than others, and the preponderance of one or more of the miasms may turn our attention to that particular group for further study.

If we fail in our selection we must not assign it to the poverty of the materia medica, or to the lack of the proving of a particular drug. Allen's *Encyclopædia* and the *Guiding Symptoms* are perfect mines of remedies and symptoms of which we know too little. There is too much materia medica rather than not enough.

In conclusion, let me say that it is exceedingly difficult to carry into actual practice the various rules and precepts that govern our most noble art of healing. When confronted with actual cases it is not the beginner alone who blunders; the most experienced will do so without apparent cause. But if we have an ideal in our art that we endeavor to attain, then our blunders and mistakes may not be useless, provided they serve as stepping-stones to a higher appreciation of Hahnemann's teachings and an increased ability to follow them.

